# 197 Association between physical activity and chronic diseases in the adult population in Slovenia

**DOI:** 10.1093/eurpub/ckae114.042

**Published:** 2024-09-26

**Authors:** Monika Brovč, Andreja Kukec, Suzana Pustivšek, Pia Vračko

**Affiliations:** National Institute of Public Health (NIJZ), Slovenia; Faculty of Medicine, University of Ljubljana, Slovenia; World Health Organization, North Macedonia

## Abstract

**Purpose:**

The purpose of this study was to examine the results of the latest European health interview survey (EHIS 2019) to analyze the prevalence of non-communicable diseases (NCDs) and the association between them and physical activity (PA) in adults in Slovenia, and to determine the role of socio-economic and other factors. The main hypothesis was that people who achieve the World Health Organization’s recommended physical activity have less diseases than those who do not. The innovative approach was the use of multivariate analysis to examine the correlations between different factors.

**Methods:**

Data from the EHIS 2019 survey was used. It is a cross-sectional study of a representative sample of adults aged 15 and over, conducted periodically in EU member states. 9900 adults participated in the Slovenian study. Chi-square test was used to assess the statistical difference between the observed outcomes and explanatory variables and binary logistic regression to assess the interdependence of all selected variables.

**Results:**

People who met PA recommendations had a lower prevalence of NCDs compared to those who did not (36.3% vs. 50.7%, p < 0.001), as well as those who met the muscle strengthening recommendations (32.2% vs. 40.7%, p < 0.001). The higher percentages of individuals meeting the recommendations were among women, younger age groups, single individuals, those who didn’t have NCDs, those sitting less than 4 hours per day, and those with a normal body mass index. In multivariate analysis, there was a significant difference between those achieving the recommended PA and those who did not; the latter had an approximately 1.4-fold higher odds ratio of having at least one NCD (p < 0.001). People who sit for 12 hours or more per day had approximately 1.8-fold higher odds ratio of having an NCD (p = 0.019).

**Conclusions:**

Based on the results of the study, it would be useful to emphasize the importance of physical inactivity and prolonged sitting for health and to strengthen public health programmes to promote PA and reduce sitting time.

**Support/Funding Source:**

This study didn’t receive funding.

